# Utility of whole-genome sequence data for across-breed genomic prediction

**DOI:** 10.1186/s12711-018-0396-8

**Published:** 2018-05-18

**Authors:** Biaty Raymond, Aniek C. Bouwman, Chris Schrooten, Jeanine Houwing-Duistermaat, Roel F. Veerkamp

**Affiliations:** 1 Animal Breeding and Genomics, Wageningen University and Research, P.O. Box 338, 6700 AH Wageningen, The Netherlands; 2 Biometris, Wageningen University and Research, 6700 AA Wageningen, The Netherlands; 3 CRV BV, P.O. Box 454, 6800 AL Arnhem, The Netherlands; 4 Department of Medical Statistics and Bioinformatics, Leiden University Medical Centre, 2333 ZC Leiden, The Netherlands; 5 School of Mathematics, University of Leeds, Leeds, LS2 9JT UK

## Abstract

**Background:**

Genomic prediction (GP) across breeds has so far resulted in low accuracies of the predicted genomic breeding values. Our objective was to evaluate whether using whole-genome sequence (WGS) instead of low-density markers can improve GP across breeds, especially when markers are pre-selected from a genome-wide association study (GWAS), and to test our hypothesis that many non-causal markers in WGS data have a diluting effect on accuracy of across-breed prediction.

**Methods:**

Estimated breeding values for stature and bovine high-density (HD) genotypes were available for 595 Jersey bulls from New Zealand, 957 Holstein bulls from New Zealand and 5553 Holstein bulls from the Netherlands. BovineHD genotypes for all bulls were imputed to WGS using Beagle4 and Minimac2. Genomic prediction across the three populations was performed with ASReml4, with each population used as single reference and as single validation sets. In addition to the 50k, HD and WGS, markers that were significantly associated with stature in a large meta-GWAS analysis were selected and used for prediction, resulting in 10 prediction scenarios. Furthermore, we estimated the proportion of genetic variance captured by markers in each scenario.

**Results:**

Across breeds, 50k, HD and WGS markers resulted in very low accuracies of prediction ranging from − 0.04 to 0.13. Accuracies were higher in scenarios with pre-selected markers from a meta-GWAS. For example, using only the 133 most significant markers in 133 QTL regions from the meta-GWAS yielded accuracies ranging from 0.08 to 0.23, while 23,125 markers with a − log10(p) higher than 7 resulted in accuracies of up 0.35. Using WGS data did not significantly improve the proportion of genetic variance captured across breeds compared to scenarios with few but pre-selected markers.

**Conclusions:**

Our results demonstrated that the accuracy of across-breed GP can be improved by using markers that are pre-selected from WGS based on their potential causal effect. We also showed that simply increasing the number of markers up to the WGS level does not increase the accuracy of across-breed prediction, even when markers that are expected to have a causal effect are included.

## Background

Genomic prediction (GP) has become a routine procedure in livestock breeding schemes [1, 2], and its adoption has resulted in a significant increase in the rate of genetic gain. This is especially evident in dairy cattle, where its application has led to a doubling of the rate of genetic gain [3], as compared to the traditional progeny-testing scheme. Genomic prediction has been very successful mainly because it enables the estimation of genomic estimated breeding values (GEBV) for young bulls, thereby drastically reducing the generation interval. This method has been implemented mainly for within-breed genetic evaluation for which both the reference and the validation sets come from the same breed.

The use of the effect of markers that are trained in one breed to obtain GEBV for animals from a different breed, a method called across-breed GP has been rather unsuccessful. Reported accuracies for across-breed GP using the genomic best linear unbiased prediction (GBLUP) model are clustered around 0 [4–7], except in rare cases in which the quantitative trait loci (QTL) that underlie the trait are known and information about these QTL is used in the prediction model [8–10]. The poor performance of across-breed GP could be due to a number of reasons, some of which include differences in linkage disequilibrium (LD) phase between markers and QTL across breeds, QTL segregating in only one breed and not the other, differences in minor allele frequencies (MAF) and allele substitution effects due to the different genetic background of the breeds [11–13]. An earlier hypothesis was that accuracies of across-breed prediction increase when higher density marker panels are used e.g., De Roos et al. [14]. An important question in this regard is how much gain in accuracy of across-breed prediction can be achieved if whole-genome sequence (WGS) markers are used instead of the lower density marker panels.

Given that multi-locus LD across breeds is conserved only over short distances [14], the expectation is that for a large proportion of the markers, LD with causal loci changes across breeds, thus they become independent of the causal loci. Our hypothesis is that independent, non-causal markers from WGS data exhibit a dilution effect on accuracy of across-breed prediction. In other words, these markers have no explanatory value and only serve as additional sources of noise in prediction models, which in turn negatively impact the accuracy of predicting GEBV across breeds. For example, in their simulation study, Wientjes et al. [15] obtained an increase in the accuracy of across-breed GP of approximately 53% by selecting only the markers that surround the causal mutations rather than using all those available, effectively eliminating the non-causal markers that are not in LD with the causal loci. Similar results were obtained by Van den Berg et al. [16]. To fully use WGS data for across-breed GP, it may become necessary to first pre-select markers with a potential causal effect on the trait of interest and use only these for prediction.

Thus, our objective was to investigate the effect of using WGS data instead of the lower density marker panels on the accuracy of across-breed genomic prediction. In addition, we assessed the importance of pre-selecting markers, using the significance and the conditional and joint (COJO) effects of markers in a meta-GWAS as selection criteria. We investigated this objective using 595 New Zealand Jersey and 957 New Zealand Holstein bulls, as well as 5553 Dutch Holstein bulls, all of which had deregressed proofs (DRP) for stature and bovine HD genotypes that were imputed to WGS.

## Methods

### Phenotype

Stature was used as a model trait in all analyses. Estimated breeding values (EBV) and number of daughters were available for 735 Jersey (NZJ) and 1239 Holstein (NZH) bulls from New Zealand and 5553 Dutch Holstein (DH) bulls. Data were provided by CRV BV (Cooperative Cattle Improvement Organization, Arnhem, the Netherlands). The EBV for stature for all bulls were deregressed to obtain deregressed proof (DRP) using the iterative procedure described in Calus et al. [17]. This approach, called matrix deregression corrects for information of the parents of an individual on its EBV and shrinkage to the mean. To avoid double-counting of information, matrix deregression also corrects for information of other relatives that are included in the same reference population as the individual whose EBV is being deregressed. The output from this procedure were the DRP and deregressed effective daughter contributions (EDC). Animals with deregressed EDC of 0 were removed, resulting in 595 NZJ, 957 NZH and 5553 DH bulls in the final dataset. Deregressed EDC were used as weights for the DRP in subsequent analyses. For the DH set, the mean EDC was equal to 52, while for the NZH and NZJ sets, it was equal to16 and 17, respectively. The set of DH individuals included in the meta-GWAS of Bouwman et al. [18] was the exact same set of DH individuals used in our study.

### Genotypes and imputation to WGS

The bulls were either genotyped with the Illumina BovineHD Bead chip (HD; 734,403 SNPs; Illumina Inc., San Diego, USA), or with a 50K single nucleotide polymorphism (SNP) chip and imputed to HD. The HD genotypes were subsequently imputed up to WGS using the sequenced population from the 1000 Bull Genomes Project Run 4 as reference population. This multi-breed reference population contained 1147 sequenced animals with on average 11-fold coverage. The reference population contained 311 Holstein and 61 Jersey animals, but all individuals were used as reference because earlier studies showed that a multi-breed sequenced reference population can be beneficial for imputation accuracy, especially for SNPs with a low minor allele frequency (MAF) [19–21]. Polymorphic sites, including SNPs and short insertions and deletions (indels) were identified in the 1147 individuals simultaneously as described in Daetwyler et al. [21]. The genotype calls of the 1000 bull genomes reference population were improved with BEAGLE 4.0 [22]. The sequence data contained 30,339,468 bi-allelic markers (SNPs and indels) with four or more copies of the minor allele in the reference population, which were used for imputation. Further details about the 1000 bull genomes reference population, variant calling and filtering of variants are in Daetwyler et al. [21].

Imputation of HD genotypes to WGS was done using standard settings in MINIMAC2 [23] and the pre-phased reference genotypes (SNPs and indels) that resulted from BEAGLE 4.0 (default settings) [22]. The imputed data were allele dosages that were subsequently converted to most probable genotypes. The output from MINIMAC2 included an r^2^ value, which is the estimated correlation between imputed and true genotypes. Markers that had an r^2^ lower than 0.5 and those that were either monomorphic or had only opposing homozygous genotypes were filtered out. We also filtered out markers that had less than 10 copies of the minor allele in the populations. This criterion was the same as setting a MAF threshold of $\frac{10}{N*2},$ where *N* is the number of genotyped individuals. Filtering based on MAF was done separately for the NZH, NZJ and DH sets. Furthermore, in the final dataset we retained only markers that segregated in all three populations.

The results of a meta-GWAS analysis conducted on 58,265 individuals with imputed WGS were used to select variants associated with stature [18]. This meta-analysis was performed on 17 populations from eight breeds, including Holstein and Jersey, from different countries. The DH population was included in the meta-GWAS, whereas both the NZH and NZJ populations were not included. The meta-analysis across the populations found 24,230 genome-wide significant (*p* < 5 × 10e–8) WGS markers in 163 distinct QTL regions that were spread across 27 autosomes (excluding chromosomes 24 and 27). For each of the 163 regions, the lead marker (160 SNPs and three indels) was determined based on its highest significance.

### Scenarios and selection of markers

In our first scenario, we used all available markers in the WGS data for GP, to test the hypothesis that the number of markers used determines the accuracy of across-breed predictions. We also created scenarios that contained only markers that showed statistical significance in the previously conducted meta-GWAS analysis to evaluate whether the non-significant markers from the meta-GWAS only add noise in a GP model. Since markers can show spurious associations based on the effect of LD and family structure, simply selecting markers based on statistical significance is not sufficient to filter out all the potentially non-causal markers. Hence, we performed a forward selection of markers to create other scenarios using the conditional and joint (COJO) effect method described in Yang et al. [24] and implemented in the GCTA software [25]. The COJO option in GCTA is a stepwise marker selection procedure that selects independently associated markers, given some parameter thresholds and the summary level result from a GWAS. As outlined in the main paper of Yang et al. [25], the marker selection model is initiated with the most significant marker that is below the threshold, followed by a calculation of the conditional *p* values for the remaining markers i.e. conditional on the marker that is already in the model. The marker with the lowest conditional *p* value is then selected and added to the model, and the process is repeated. It is possible that the marker being tested is in high LD with other markers that are already in the model. To avoid having such markers in the model, a collinearity threshold is set. If the threshold is exceeded, the marker being tested is dropped and the next one is considered instead. A window size in mega base pairs (Mb) is defined, outside of which markers are assumed to be in linkage equilibrium. Once all the markers have been considered and those that are independently significant have been identified by the model and selected, the selected markers are then jointly fitted in a model and those with a p value higher than the threshold are dropped. These steps are repeated iteratively until no more markers can be added or removed from the model. In total, 10 scenarios were created depending on whether or not variants were pre-selected from WGS data and the criteria of selection that were used. All the scenarios are described in Table 1. The total number of markers in each scenario and their mean MAF are in Table 2.

**Table 1:** Description of scenarios used in the study

Scenario	Description
*Scenarios with unselected markers*	
Full_seq	All available markers in WGS data that met the quality check criteria were included
HD	SNPs on the commercial BovineHD SNP chip
50k	SNPs on the commercial 50k chip
*Scenarios with selected marker *+* unselected markers*	
HD_Top	Top_markers added to HD
50k_Top	Top_markers added to 50k
*Scenarios with only selected markers*	
Pval5	All markers that had a − log10(p) value higher than 5 from the meta-GWAS analysis
Pval7	All markers that had a − log10(p) value higher than 7 form the meta-GWAS analysis
COJO3	Markers selected using the following COJO model parameters thresholds: conditional and joint *p* value threshold (p) = 5e–3, collinearity between selected markers = 0.9, window size = 10 Mb
COJO8	Markers selected using the following COJO model parameters thresholds: conditional and joint p-value threshold (p) = 5e–8, collinearity between selected markers = 0.9, window size = 10 Mb
Top_markers	The most significant markers identified in each defined QTL region from the meta-GWAS analysi; only those that passed QC are included here

**Table 2:** Number of markers in each scenario and their mean minor allele frequency in Dutch Holsteins (DH), New Zealand Holsteins (NZH) and New Zealand Jerseys (NZJ)

Scenario	Number of SNPs	Mean MAF		
		DH	NZH	NZJ
*Scenarios with unselected SNPs*				
Full_seq	14,341,737	0.18	0.17	0.15
HD	583,078	0.27	0.28	0.24
50k	48,912	0.25	0.26	0.21
*Scenarios with unselected *+* selected SNPs*				
HD_TOP	583,194	0.27	0.28	0.24
50k_TOP	49,045	0.25	0.25	0.21
*Scenarios with only selected SNPs*				
Pval5	59,828	0.25	0.23	0.17
Pval7	23,125	0.24	0.23	0.17
COJO3	1570	0.18	0.17	0.13
COJO8	360	0.20	0.21	0.13
Top_markers	133	0.23	0.24	0.16

### Proportion of genetic variance explained

In each scenario, a genomic relationship matrix (**GRM**) was calculated for all the animals according to the first method of VanRaden [26]. This was done using calc_grm [27] as:

$${\mathbf{GRM}} = \frac{{{\mathbf{ZZ^{\prime}}}}}{{2\sum p_{j} \left( {1 - p_{j} } \right)}},$$

where *p*_*j*_ is the frequency of the allele of marker *j* with the homozygous genotype being coded as 2, and **Z** is the centred (*Z*_*ij*_ = *X*_*ij*_ − 2*p*_*j*_) genotype matrix. $X_{ij}$ is the genotype of marker *j* for animal *i*, with the elements of *X*_*ij*_ coded as 0, 1, 2. Using the estimated **GRM** and the DRP of the reference animals as phenotypes, a genomic restricted maximum likelihood (GREML) analysis was run in ASReml [28] to estimate the proportion of genetic variance captured by the markers in each scenario. In the GREML model, deregressed EDC were used to scale the residual of each animal in the model such that animals with a high deregressed EDC has effectively a small residual. The weighting procedure is described in more detail below in the genomic prediction sub-section. Normally, the residual variance obtained from such analyses is defined for a weight of 1, but because the deregressed EDC that were used as weights were considerably higher than 1, the resulting residual variances were inflated. To correct for this, we scaled the residual variances by the mean of the deregressed EDC in the data [29]. For example, if the mean of deregressed EDC in the data is *k*, we computed the phenotypic variance as: $var\left( {\mathbf{g}} \right) + \frac{{var\left( {\mathbf{e}} \right)}}{k},$ where $var\left( {\mathbf{g}} \right)$ is the genetic variance and $var\left( {\mathbf{e}} \right)$ is the residual variance.

### Genomic prediction with markers in the different scenarios

For GP, the composition of reference and validation sets are in Table 3. Estimation of GEBV and the proportion of genetic variance captured by markers as explained in the previous section was carried out simultaneously using the same **GRM**, phenotype data and model. A single-trait animal model was fitted in ASReml [28] as follows:

$${\mathbf{y}} = {\mathbf{1}}\upmu + {\mathbf{Wg}} + {\mathbf{e}},$$

where ${\mathbf{y}}$ is a vector containing stature DRP for the reference set (missing for the validation set), **1**μ is the mean of the DRP, ${\mathbf{g}}$ is a vector of additive genetic effects for all animals, ${\mathbf{W}}$ is the design matrix that links ${\mathbf{g}}$ to the DRP in ${\mathbf{y}}$ and ${\mathbf{e}}$ is a vector containing the residuals. Both ${\mathbf{g}}$ and ${\mathbf{e}}$ are assumed to be normally distributed as ${\mathbf{g}} = N\left( {{\mathbf{0}},{\mathbf{GRM}}\upsigma_{\text{g}}^{2} } \right)$ and ${\mathbf{e}} = N\left( {{\mathbf{0}},{\mathbf{D}}\upsigma_{\text{e}}^{2} } \right),$ where $\upsigma_{\text{g}}^{2}$ the genetic variance, ${{\upsigma }}_{\text{e}}^{2}$ the residual variance and ${\mathbf{D}}$ is a diagonal matrix that contains the inverse of deregressed EDC, which in this case were used as weights for the DRP in the model. We measured the accuracy of GP as the correlation between the GEBV and the DRP of the validation animals. The standard error (SE) of accuracy was calculated based on the sampling variance of a correlation as ${\text{SE}} = \frac{{1 - r^{2} }}{{\sqrt {N - 2} }}$, where *r* is the accuracy and *N* is the sample size (number of validation candidates).

**Table 3:** Composition of reference and validation sets used in the study

Reference set (size)	Validation set (size)
New Zealand Holsteins (957)	New Zealand Jerseys (595)
	Dutch Holsteins (5553)
New Zealand Jerseys (595)	New Zealand Holsteins (957)
	Dutch Holsteins (5553)
Dutch Holsteins (5553)	New Zealand Holsteins (957)
	New Zealand Jerseys (595)

Furthermore, prediction bias was assessed by regressing DRP of validation animals on their GEBV. Stature is measured on a different scale in New Zealand and in the Netherlands. As a result, the GEBV of the NZH and NZJ bulls that were obtained by using a DH reference population will be on the DH scale, while their DRP are on a different scale. To estimate the slope and intercept of regression, we rescaled the DRP and GEBV so that they are on the same scale and expressed in their standard deviation units as:

$$\begin{aligned} {\text{Scaled}}\_{\text{DRP}} = \frac{1}{{\left( {{\text{SD}}_{\text{DRP}} } \right)}}*({\text{DRP}}\text{ - }{\overline{\text{DRP}}}), \\ {\text{Scaled}}\_{\text{GEBV}} = \frac{1}{{\left( {{\text{SD}}_{{{\text{DRP}}\_{\text{Ref}}}} } \right)}}*\left( {\text{GEBV}} \right), \\ \end{aligned}$$

where ${\text{Scaled}}\_{\text{DRP}}$ and ${\text{Scaled}}\_{\text{GEBV}}$ are the rescaled DRP and GEBV of the validation animals, ${\text{DRP}}$ is the DRP of the validation animals on their original scale, ${\text{SD}}_{\text{DRP}}$ is the standard deviation of ${\text{DRP}}$ and ${\text{SD}}_{{{\text{DRP}}\_{\text{Ref}}}}$ is the standard deviation of the DRP of reference population.

## Results

### Proportion of genetic variance explained

**Fig. 1:**
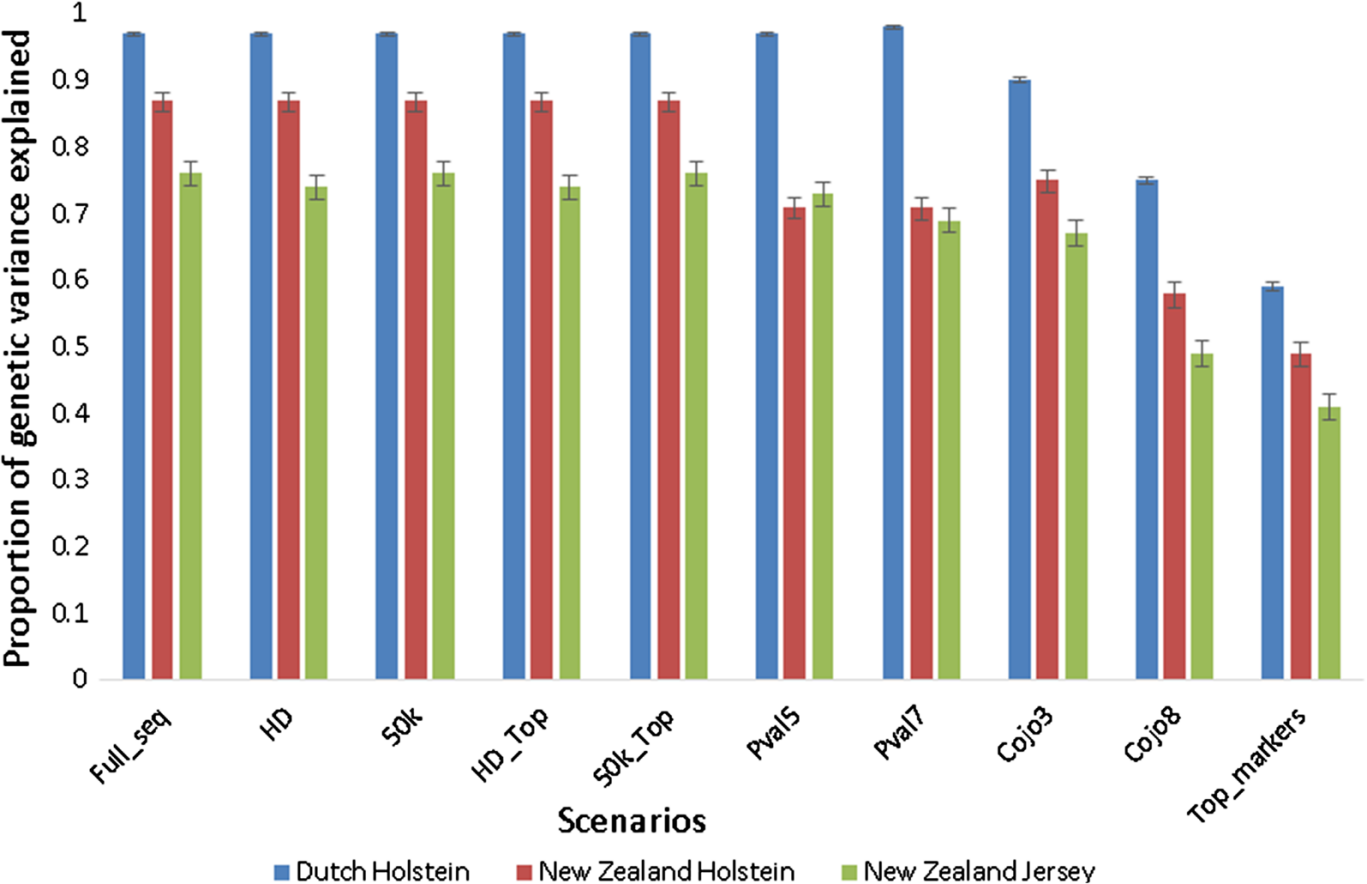
Proportion of genetic variance captured by markers in the different scenarios. The standard errors are indicated at the top of each bar

We estimated the proportion of genetic variance captured by markers in the different scenarios. This was done separately for the DH, NZH and NZJ datasets and the results are presented in Fig. 1. Across the three populations, the proportion of genetic variance captured increases almost linearly with the number of markers up to 50k. Above 50k, the proportion of genetic variance captured plateaus. Consequently, using WGS markers did not lead to any significant gain in the proportion of genetic variance captured compared to the lower density panels.

Except for the extreme cases of the Top-markers, COJO8 and COJO3 scenarios that contained only 133, 360 and 1570 markers, respectively, the proportion of genetic variance captured did not differ markedly between scenarios. With only 133 markers that were identified as lead markers in a meta-GWAS analysis [18] and across the three populations, 41 to 59% of the genetic variance was captured, which is more than half of the genetic variance captured by the WGS data, which include approximately 14 million markers (76 to 97%). Across all the scenarios, more genetic variance was captured in the DH population followed by the NZH population. This was expected, given that the DH bulls were included in the meta-GWAS that was used to select the markers, whereas the New Zealand populations were not included. Moreover, about 40% of the animals used for the meta-GWAS were Holsteins whereas only about 5% of the animals were Jerseys from Australia. Another important reason in this regard is the large size of the DH population compared to those of NZH and NZJ.

### Accuracy of across-breed genomic prediction

Our main objective was to investigate how much gain in accuracy of genomic prediction can be achieved across breed and across country by using WGS data instead of the lower density marker panels. As presented in Table 4, using WGS data did not lead to any gain in the accuracy of GP compared to using HD or 50k markers. Accuracy of prediction was close to 0 (0.02 to 0.08) in the across-breed, across-country (DH and NZJ) GP scenario. Similarly, accuracy of prediction obtained using WGS data ranged from 0.06 to 0.09 in the across-breed but within-country (NZH and NZJ) GP scenario. In the within-breed, across-country (NZH and DH) GP scenario, accuracies of prediction were considerably higher, ranging from 0.41 to 0.51. However, even in this case the accuracies obtained using WGS data were lower than those obtained using the 50k and HD marker panels.

**Table 4:** Accuracies of prediction for all prediction scenarios in the study

	Across-breed, across-country		Across-breed, within-country		Within-breed, across-country	
Reference	DH	NZJ	NZH	NZJ	NZH	DH
Validation	NZJ	DH	NZJ	NZH	DH	NZH
*Scenarios with unselected markers (number of markers)*						
Full_seq (14,341,737)	0.08	0.02	0.09	0.06	0.41	0.51
HD (583,078)	0.10	0.05	0.07	− 0.04	0.43	0.55
50k (48,912)	0.06	0.05	0.13	− 0.04	0.42	0.52
*Scenarios with unselected markers *+* selected markers (number of markers)*						
HD_Top (583,194	0.10	0.05	0.07	− 0.04	0.43	0.55
50k_Top (49,045)	0.07	0.06	0.13	− 0.03	0.43	0.53
*Scenarios with only selected markers (number of markers)*						
Pval5 (59,828)	0.19	0.19	0.14	0.32	0.52	0.58
Pval7 (23,125)	0.01	0.15	0.07	0.35	0.46	0.47
COJO3 (1570)	0.20	0.22	0.14	0.31	0.45	0.52
COJO8 (360)	0.21	0.25	0.16	0.18	0.47	0.44
Top_markers (133)	0.23	0.18	0.08	0.27	0.41	0.47
SE*	0.04	0.01	0.04	0.03	0.01	0.01

Predictions were carried out either across breed and across country, across breed and within country or within breed and across country. The populations are Dutch Holsteins (DH), New Zealand Holsteins (NZH) and New Zealand Jerseys (NZJ)

The unselected marker sets are the whole-genome sequence (Full_seq), high-density markers (HD) and markers on the traditional 50k chip (50k). HD_Top and 50k_Top are scenarios in which some pre-selected markers from a meta-GWAS (Top_markers) are added to the HD and 50k markers respectively. Pval5 and Pval7 are marker sets pre-selected from a meta-GWAS based on their p values. The COJO scenarios are those containing markers that are assumed to be independently significant markers from a meta-GWAS at different significant levels. Top_markers contain the most significant markers in each QTL region from a meta-GWAS

*Standard error (SE) of estimates, did not differ across the different sets of markers, provided the reference and validation populations remained the same

The remaining scenarios, apart from those with WGS data, which contained unselected markers (50k and HD), also performed poorly in across-breed prediction with accuracies ranging from − 0.04 to 0.13. The Top_markers were subsequently added to the 50k and HD markers to form new scenarios (50k_Top and HD_Top, respectively). However, using the combined marker sets (50k_Top and HD_Top) in across-breed GP resulted in low accuracies of prediction (− 0.04 to 0.13), similar to those obtained by using only the 50k and HD markers. Using the 50k_Top and HD_Top markers in within-breed GP, but across countries, also resulted in similar accuracies as those obtained by using only 50k and HD. Overall, adding the potentially causal markers to the 50k and HD markers did not result in any gain in the accuracy of GP.

The prior-selection of markers from WGS data, which was based on their potential causal effect, and the benefit of using such markers for across-breed GP were also investigated in this study. Accuracies of across-breed prediction obtained by using the pval5 markers ranged from 0.14 to 0.32, whereas accuracies obtained with the pval7 markers ranged from 0.07 to 0.35. Accuracies of prediction using the Pval5 and Pval7 markers are already 3 to 4 times higher than those obtained using WGS data, although they contained only very small fractions of the number of markers that are in WGS data. In the within-breed, across-country GP scenario, we also observed a slight increase in the accuracy of GP using pval5 and pval7 markers compared to all the scenarios with unselected markers.

One can argue that selecting markers purely on significance in a GWAS is naive, in the sense that some of these markers are selected simply because they are in LD with causal mutations and that they provide redundant information in GP. It was for this reason that we performed forward marker selection to limit the effect of LD, thus resulting in the COJO3 and COJO8 scenarios. The COJO scenario contained independently significant markers from the meta-GWAS at a significance level of 5e–3 for COJO3 and 5e–8 for COJO8. Accuracy of across-breed prediction using the markers in the COJO3 scenario ranged from 0.14 to 0.31, whereas that in the COJO8 scenario ranged from 0.16 to 0.25. This indicates that the COJO scenarios have about the same level of accuracy as the Pval scenarios, in spite of having fewer markers. The accuracy of across-breed prediction using only the 133 top markers from the meta-GWAS ranged from 0.08 to 0.27, which for some across-breed prediction scenarios (NZJ as reference population or DH as reference population to predict GEBV for NZJ) is approximately 3 times higher than the accuracy obtained using WGS data.

### Prediction bias

In this study, we assessed prediction bias based on the slope of regression when the DRP of the validation animals were regressed onto their GEBV; the results are in Table 5. For across-breed, across-country GP, the slope ranged from 0.01 to 0.81, and there was no apparent difference between the scenarios with selected and unselected markers sets. For across-breed, within-country GP, predictions were also biased. GEBV for the NZJ predicted from the NZH were underestimated, whereas GEBV for the NZH predicted from the NZJ were overestimated. Exceptions were the scenarios HD, HD_Top, 50k and 50k_Top, which all had negative slopes, although these estimates had high standard errors and were not significantly different from zero. These scenarios using unselected marker sets, also had close to zero (and slightly negative) prediction accuracies (Table 4). For within-breed, across-country GP, when DH was used as reference population to predict GEBV for NZH, slopes deviated more from 1 in the scenarios with pre-selected markers (0.55–0.72) than those with unselected markers (0.77–0.85). When GEBV for DH were predicted from NZH, the use of pre-selected markers or unselected markers made a difference in either over- or under-predicting the breeding values.

**Table 5:** Intercept and slope of regression when deregressed breeding values of validation animals were regressed onto their predicted genomic breeding values

Across-breed, across-country				
Scenarios	DH reference, NZJ validation		NZJ reference, DH validation	
	Intercept	Slope	Intercept	Slope
Full_seq	0.28	0.38 (0.20)	− 0.01	0.36 (0.22)
HD	0.39	0.41 (0.16)	− 0.02	0.73 (0.20)
50k	0.24	0.20 (0.14)	− 0.01	0.63 (0.16)
HD_Top	0.39	0.41 (0.16)	− 0.02	0.74 (0.20)
50k_Top	0.28	0.23 (0.13)	− 0.02	0.73 (0.16)
Pval5	0.30	0.36 (0.08)	− 0.03	0.59 (0.04)
Pval7	0.01	0.01 (0.06)	− 0.04	0.42 (0.03)
COJO3	0.40	0.35 (0.07)	− 0.05	0.71 (0.04)
COJO8	0.13	0.40 (0.07)	− 0.03	0.81 (0.04)
Top_markers	0.46	0.43 (0.08)	− 0.06	0.48 (0.03)

Across-breed, within-country				
Scenarios	NZH reference, NZJ validation		NZJ reference, NZH validation	
	Intercept	Slope	Intercept	Slope
Full_seq	0.37	1.17 (0.01)	− 0.04	0.87 (0.47)
HD	0.47	1.17 (0.01)	0.02	− 0.58 (0.43)
50k	0.45	1.17 (0.01)	0.02	− 0.43 (0.36)
HD_Top	0.47	1.17 (0.01)	0.02	− 0.57 (0.43)
50k_Top	0.46	1.19 (0.01)	0.02	− 0.33 (0.36)
Pval5	0.32	1.19 (0.02)	0.01	0.90 (0.08)
Pval7	0.40	1.16 (0.03)	0.05	0.87 (0.07)
COJO3	0.52	1.20 (0.02)	0.05	0.89 (0.09)
COJO8	0.54	1.13 (0.03)	0.00	0.56 (0.10)
Top_markers	0.51	1.02 (0.04)	0.03	0.70 (0.08)

Within-breed, across-country				
Scenarios	DH reference, NZH validation		NZH reference, DH validation	
	Intercept	Slope	Intercept	Slope
Full_seq	0.45	0.85 (0.04)	− 0.24	1.16 (0.03)
HD	0.40	0.81 (0.04)	− 0.27	1.17 (0.03)
50k	0.36	0.77 (0.04)	− 0.27	1.13 (0.03)
HD_Top	0.40	0.81 (0.04)	− 0.27	1.17 (0.03)
50k_Top	0.38	0.78 (0.04)	− 0.27	1.14 (0.03)
Pval5	0.47	0.72 (0.03)	− 0.27	1.09 (0.02)
Pval7	0.24	0.55 (0.03)	− 0.27	0.98 (0.03)
COJO3	0.44	0.64 (0.03)	− 0.22	0.91 (0.02)
COJO8	0.33	0.55 (0.04)	− 0.22	0.91 (0.02)
Top_markers	0.47	0.69 (0.04)	− 0.16	0.74 (0.02)

The populations are Dutch Holsteins (DH), New Zealand Holsteins (NZH) and New Zealand Jerseys (NZJ). Standard errors for the slopes are given in parentheses

The unselected marker sets are the whole-genome sequence (Full_seq), high-density markers (HD) and markers on the traditional 50k chip (50k). HD_Top and 50k_Top are scenarios in which some pre-selected markers from a meta-GWAS (Top_markers) are added to the HD and 50k markers, respectively. Pval5 and Pval7 are marker sets pre-selected from a meta-GWAS based on their p values. The COJO scenarios are those containing markers that are assumed to be independently significant markers from a meta-GWAS at different significant levels. Top_markers contain the most significant markers in each QTL region from a meta-GWAS

## Discussion

The objective of this study was to investigate the potential gain in accuracy of across-breed prediction when WGS data are used instead of lower density marker panels. Our results showed that simply increasing the number of markers up to WGS does not result in an increased accuracy of across-breed prediction and that, in some cases, it can even result in lower accuracies compared to when 50k or HD marker panels are used. These results are in agreement with previous studies that showed no added benefit of using WGS data for GP [30–33], although in these studies, GP was carried out within breed.

One of the explanations for the low accuracy of across-breed GP is the inconsistency of LD between markers and causal mutations across breeds [34]. On the one hand, this challenge can be circumvented by simply increasing the number of markers up to the point that each causal locus is in LD with at least one marker across breeds. For example, in cattle it was shown that about 300,000 SNPs are sufficient to ensure the consistency of LD phase across breeds [14], which is far less than the 14 million markers that we used in this study. On the other hand, increasing the number of markers up to WGS increases the chance that causal mutations are in LD with a large number of non-causal markers, even across breeds. Thus, the effect of the causal markers are picked up by a linear combination of many other non-causal markers, with which the causal markers are in LD. In other words, with a GREML model, the effect of the causal markers is shrinked towards the mean to obtain a uniform distribution of marker effects, as is the assumption in the infinitesimal model. As a result, the model is not able to pinpoint the true effect of the causal markers across breeds, thereby resulting in low accuracies of prediction.

Another possible explanation for our result, which was also put forward by Wientjes et al. [15], is that the accuracy of estimating marker effects is low. The low accuracy of estimating marker effects can be due to differences in allele substitution effects at the causal loci between the breeds [12], resulting from differences in genetic background, differences in MAF between breeds, possible gene-by-environment interactions, different contributions of non-additive gene effects and the limited size of available data. The differences in allele substitution effects at the causal loci reflect the genetic correlation between breeds [35, 36], which in theory sets an upper limit for the accuracy of across-breed GP. Although the low accuracies that we observed for across-breed prediction might have been exacerbated by the low accuracies of estimating marker effects, we believe that another major underlying reason is the increased model complexity and over-parameterization that comes along with the use of WGS data in GP.

In this study, we investigated the effect of prior selection of markers from a meta-GWAS study on the accuracy of across-breed GP. Our results clearly demonstrated that when markers in the WGS data are selected a priori based on their potential causal effect and that the potentially non-causal markers are excluded, the accuracy of across-breed prediction can be increased significantly. We believe that the non-causal markers in WGS data exhibit some kind of dilution effect on the accuracy of genomic prediction. Although a slight dilution effect of non-causal markers was noted for within-breed, across-country GP i.e., when we used DH to predict NZH and the other way round, this effect was more pronounced in an across-breed GP scenario. A possible explanation is that within breeds, LD extends over long distances, which increases the chance that every marker in the dataset is in LD with a causal locus and with other markers in LD with the same causal locus. Thus, many of the markers, although having no causal effect on their own, still have an explanatory value due to LD. However, LD between markers and causal loci across breeds is conserved only across short distances [14]. This means that across breeds, there are probably many markers that are not linked to any causal locus, i.e., they have different LD patterns in different breeds, which in a way make them serve as sources of noise and dilution factors in across-breed GP. The proportion of these ‘lone standing’ markers and the corresponding noise effect increase when for example instead of 50k or HD marker panels, WGS data are used for GP across distantly related breeds. Our results agree completely with those of van den Berg et al. [16] who found in their simulation studies that GP using markers that are in LD with causal loci was more accurate than when all markers (both those in LD with causal loci and those that are not) were used together. Similar results were obtained by Brondum et al. [20]. We have empirically shown that there is a good prospect for the use of WGS data in across-breed GP, provided the potentially causal markers in such data are accurately identified and used for prediction.

In this study, we benefitted from the results of a very powerful meta-GWAS [18]. Thus, there is a high level of confidence that the pre-selected markers from the meta-GWAS are either the causal variants themselves or are in high LD with the causal variants. This explains the significant improvement in accuracies of across-breed GP resulting from the use of pre-selected markers compared to the use of unselected marker sets (Table 4). Such a powerful meta-GWAS for pre-selection of markers is currently rather unique, hence pre-selection of markers for other traits is not a trivial task. Other ways to pre-select markers from whole-genome sequence based on their significance for the trait of interest include GWAS using (a subset of) the reference population or variable selection models. Performing a GWAS using data on the reference population and using preselected markers based on their significance from the GWAS for GP in the same population was shown to be less optimal than using unselected markers sets for GP [37]. Veerkamp et al. [37] found that the use of pre-selected markers from a GWAS for GP in the same population that was used for the GWAS resulted in lower accuracy of prediction and increased prediction bias compared to the use of unselected marker sets. If the use of such pre-selected markers results in decreased accuracy of within-breed prediction, the expectation is that using such markers for across-breed GP will result in little to no benefit, given that there may be differences in the properties of the markers across breeds.

In theory, similar or better prediction accuracies than those reported in this study can be obtained if data on all the individuals in the meta-GWAS are available, and if they are combined in a single multi-breed reference population to predict the GEBV of validation animals, using a Bayesian variable selection model. There are, however, two major limitations to this approach, the first being the willingness of all parties involved to share both phenotype and genotype data. In the meta-GWAS, this limitation was non-existent because the partners in the consortium shared only the summary level results of their ‘in-house’ GWAS [18] and did not have to share their phenotype or genotype data. Second, implementing a Bayesian variable selection model with 58,265 individuals all of which have WGS data is computationally not practical [31, 32].

Alternatively, a Bayesian variable selection model can be used to analyse the WGS data of the reference population in this study and the results could be applied for across-breed GP. However, with Bayesian variable selection models, like most other models, inference about marker effects or variances depends highly on sample size [38, 39], i.e., the size of the reference population. In that sense, the inferential power or accuracy of a Bayesian variable selection model using a training population of at most 5553 individuals is expected to be much lower compared to that of a meta-GWAS carried out using 58,265 individuals with WGS. Moreover, with a single breed reference population, strong family relationships and long stretches of LD can hinder the accurate pinpointing of the causal regions underlying complex traits, irrespective of the type of model applied [31, 40, 41]. For example, Habier et al. [40] showed that, in the presence of LD, prediction accuracies obtained using different models including BayesB decayed rapidly across generations. If the argument holds that such a decay in accuracy is a result of differences in LD patterns across generations, then difference in LD patterns across breeds will hinder the accurate prediction of GEBV across breeds using a single breed reference population, even with a Bayesian variable selection model.

In GP, prediction bias is commonly estimated as the regression coefficient (β) when DRP of validation animals are regressed onto their GEBV. The expectation of 1 for β does not hold in an across-breed GP scenario, mainly because the prediction model does not take all the uncertainty into account i.e. differences in LD phase between breeds and differences in allele substitution effect at the causal locus (genetic correlation less than 1) between breeds [16]. When markers are in high LD with causal mutations within the reference population, their estimated effects are likely to be close to the true effect of the causal loci, at least proportional to the extent of LD with the causal loci [42]. However, if the LD between these markers and the causal mutation does not hold across breeds, then these markers lose their effect across breeds. This loss in the effect of markers across breeds is not accounted for in the prediction model and therefore can result in the inflation of the variance of GEBV of the validation animals [43], and thus in lower slope values, as observed in most of the scenarios in our study (Table 5).

The DH population used in our study was included in the meta-GWAS that was used to pre-select the markers. When the DH population was used as a reference population to predict the GEBV of another Holstein population (NZH), predictions were more biased downwards in the scenarios with pre-selected markers than in the scenarios with unselected markers. One of the reasons for this might be that the markers are discovered in (partly) the same population as the one used to train the effects of the markers for GP, resulting in an overestimation of the effects of the markers and consequently of the variance of GEBV, a phenomenon commonly referred to as the “Beavis effect” [44]. For example, the variance of the GEBV estimated by using WGS data was equal to 0.36, while it was 0.46 when the pre-selected top-markers were used instead. Moreover, the DH and NZH populations are related, since some of the DH individuals were used as sires for the NZH population. Therefore, using DH as a reference population to predict the GEBV of NZH could have resulted in prediction error covariance between the DRP and GEBV of the validation animals [45], hence the biased predictions. Veerkamp et al. [37] also showed that predictions were more biased when they used pre-selected markers from WGS data based on the result of a GWAS, than when unselected marker sets were used. Moreover, in their study, the discovery (GWAS) population was the same as the reference population used for GP.

When GEBV are estimated with low accuracy, they are strongly regressed towards the mean, resulting in a narrow range of values for GEBV, which means that the slope of regression (bias) becomes very sensitive to even slight changes in GEBV between scenarios. For example, when the numerically small NZJ population was used as a reference population to predict GEBV for NZH, the slope of regression drastically changed from 0.87 in Full_seq to − 0.58 in HD. These scenarios have very low prediction accuracies (Table 4) and very narrow ranges of values for GEBV (− 0.22 to 0.26 for Full_seq and − 0.26 to 0.29 for HD). Because the DRP remained the same between the scenarios (− 3.37 to 3.2), only a slight change in GEBV between these scenarios could have resulted in such a huge difference in the estimated slope, accompanied by high standard errors (Table 5).

With higher accuracies, for example in the scenarios with pre-selected markers, the GEBV become less regressed to the mean compared to the scenarios with very low prediction accuracies. For example, GEBV ranged from − 1.26 to 1.33 when using the Top-markers and from − 1.1 to 1.1 when using the COJO8 markers. As a result, the slopes are estimated more precisely in these scenarios, i.e., with low standard errors and the slopes do not change drastically between the scenarios (Table 5). An important message from our results is that expectation for the bias is not 1, when across-breed differences are not properly accounted for, especially when a numerically small reference population is used. Furthermore, with low accuracies of across-breed prediction, the range of GEBV for the validation animals becomes very small due to shrinkage to the mean, and estimated bias in such cases can be very unreliable, since they are estimated with high standard errors.

## Conclusions

The use of whole-genome sequence data holds the potential to increase the accuracy of across-breed GP because it is expected to contain all the causal mutations that underlie the traits of interest. Contrary to common belief [46, 47], our results showed that simply using all markers in the WGS data in a linear GREML model results in a very low accuracy of across-breed GP. In addition to possible differences in allele substitution effects at the causal loci between breeds, this could be due to the increased model complexity and over-parameterization that accompany the use of WGS data. Furthermore, the results of this study showed that the problem of over-parametrization can be avoided partly by prior selection of markers in WGS data to contain only those with a potential causal effect. This approach enables the model to focus directly on the important regions and can result in an increase in accuracy of across-breed prediction that is more than twice that obtained by using 50k and HD marker panels or unselected WGS data. However, for some scenarios in our study, accuracies of prediction were still low even when doubled by the use of pre-selected markers instead of WGS.
